# Wnt/β-catenin and FGF signalling direct the specification and maintenance of a neuromesodermal axial progenitor in ensembles of mouse embryonic stem cells

**DOI:** 10.1242/dev.112979

**Published:** 2014-11-15

**Authors:** David A. Turner, Penelope C. Hayward, Peter Baillie-Johnson, Pau Rué, Rebecca Broome, Fernando Faunes, Alfonso Martinez Arias

**Affiliations:** Department of Genetics, University of Cambridge, Cambridge CB2 3EH, UK

**Keywords:** Wnt signalling, Mesodermal, Morphogenesis, Neural, Stem cells

## Abstract

The development of the central nervous system is known to result from two sequential events. First, an inductive event of the mesoderm on the overlying ectoderm that generates a neural plate that, after rolling into a neural tube, acts as the main source of neural progenitors. Second, the axial regionalization of the neural plate that will result in the specification of neurons with different anteroposterior identities. Although this description of the process applies with ease to amphibians and fish, it is more difficult to confirm in amniote embryos. Here, a specialized population of cells emerges at the end of gastrulation that, under the influence of Wnt and FGF signalling, expands and generates the spinal cord and the paraxial mesoderm. This population is known as the long-term neuromesodermal precursor (NMp). Here, we show that controlled increases of Wnt/β-catenin and FGF signalling during adherent culture differentiation of mouse embryonic stem cells (mESCs) generates a population with many of the properties of the NMp. A single-cell analysis of gene expression within this population reveals signatures that are characteristic of stem cell populations. Furthermore, when this activation is triggered in three-dimensional aggregates of mESCs, the population self-organizes macroscopically and undergoes growth and axial elongation that mimics some of the features of the embryonic spinal cord and paraxial mesoderm. We use both adherent and three-dimensional cultures of mESCs to probe the establishment and maintenance of NMps and their differentiation.

## INTRODUCTION

The nervous system comprises a cellular network that processes sensory and motor information to generate patterns of activity and behaviour in the organism. At the centre of this network there is a large number of interconnected neurons that shape behaviours. In mammals, the basic framework of the network is engineered during development from a sheet of neuroepithelial progenitors that arise in an anteroposterior sequence as the body plan unfolds. This process can be followed through the expression of members of the Sox2b family of transcription factors (Kamachi and Kondoh, 2013): Sox1, Sox2 and Sox3 (Pevny et al., 1998; Graham et al., 2003).

Classical studies by Spemann and Mangold (Spemann and Mangold, 1924; translated by Hamburger, 2001) showed that the emergence of the nervous system requires an inductive event mediated by a special group of cells, called the ‘organizer’, on a layer of overlying neuroectodermal cells (Spemann and Mangold, 1924; De Robertis et al., 2000; De Robertis, 2009). Later, experiments with *Xenopus* animal caps showed that the inductive event is associated with an antagonism of BMP signalling and, by extension, that BMP acts as a pan-neural repressor across the ectoderm (Hemmati-Brivanlou and Melton, 1997a). Expression of the BMP antagonists chordin, noggin and follistatin from the organizer releases this repression locally and allows the dynamic emergence of neural progenitors (reviewed by Hemmati-Brivanlou and Melton, 1997b; De Robertis et al., 2001). However, the notion that BMP antagonism is the main mechanism of neural induction has been challenged; in particular, it has been suggested that other signalling pathways, FGF/ERK and Wnt, are involved in the acquisition of the neural fate independently of the inhibition of BMP (Stern, 2005).

An important feature of the nervous system is its specialization along the anteroposterior axis, which is most obvious in the structural and functional differences between the fore-, mid- and hindbrain, and the spinal cord. The emergence of these differences is thought to occur in two steps. According to the ‘activation/transformation’ hypothesis (Nieuwkoop et al., 1952; Nieuwkoop and Nigtevecht, 1954) the neural plate is first specified, presumably by BMP antagonism, with anterior characteristics; subsequently, posterior fates, including those in the spinal cord, emerge by the action of a gradient of one or more signalling molecules (Mangold, 1933; Nieuwkoop et al., 1952; Saxén and Toivonen, 1961; reviewed by Stern et al., 2006). Experiments in *Xenopus* have led to the suggestion that the transformative influence is provided by a gradient of Wnt/β-catenin signalling (Kiecker and Niehrs, 2001). In contrast to the situation in frogs, where fates are assigned on an existing neural plate, there is evidence that in amniotes the development of the cranial and hindbrain regions, and of the spinal cord, are temporally and spatially separate (reviewed by Wilson et al., 2009; Kondoh and Takemoto, 2012). While the anterior central nervous system emerges from a neuroectodermal progenitor population following neural induction (reviewed by Andoniadou and Martinez-Barbera, 2013), work in chickens and mice has shown that the spinal cord is derived from a specialized self-renewing precursor population located within the growing caudal end of the embryo (Brown and Storey, 2000; Mathis and Nicolas, 2000; Mathis et al., 2001; Cambray and Wilson, 2002, 2007; Delfino-Machin et al., 2005; Tzouanacou et al., 2009; Nowotschin et al., 2012). Cells within this population exhibit some features of stem/progenitor cells (Mathis and Nicolas, 2000; Roszko et al., 2007; Tzouanacou et al., 2009) and can give rise to mesodermal and neural progenitors of different posterior axial levels (Brown and Storey, 2000; Cambray and Wilson, 2002; Tzouanacou et al., 2009; Tsakiridis et al., 2014). These cells, named long-term axial neuromesodermal precursors (NMp), can be identified because they co-express markers of the primitive streak (Bra) and neuroectoderm (Sox2), as well as the homeobox gene Sax1/Nkx1.2 (herein referred to as Nkx1.2), which is characteristic of the caudal neural plate (Storey et al., 1998; Delfino-Machin et al., 2005; Cambray and Wilson, 2007). Although it is possible to understand this population in the context of the activation-transformation hypothesis (Stern et al., 2006), it is also possible that it emerges *de novo* within the primitive streak as a population that has never adopted a neural plate identity. Analysis of the regulatory region of the *Sox2* gene lends some support for this possibility with the identification of an enhancer in the *Sox2* gene that drives its expression in the extending caudal neural plate, also referred to as the stem cell zone or the caudal-lateral epiblast (Wilson et al., 2009), within the progenitor population under the control of Wnt and FGF signalling (Takemoto et al., 2006, 2011; Kamachi et al., 2009).

Embryonic stem cells (ESCs) can be used as an experimental system to probe the mechanisms that mediate the specification and differentiation of cell populations in development. Recent analysis of the behaviour of epiblast stem cells (EpiSCs) in response to Chiron, an agonist of Wnt signalling, has revealed the emergence of a small population of cells that co-expresses markers of the primitive streak and Sox2, a blueprint of the NMp (Tsakiridis et al., 2014). Here, we extend these observations to mouse ESCs (mESCs) and show that modulation of Wnt signalling during controlled differentiation of mESCs in adherent culture generates a transient NMp population in an effective and reproducible manner. These progenitors require FGF signalling and can be coaxed to differentiate into neural progenitors and paraxial mesoderm with thoracic identities. Furthermore, using a recently developed 3D culture system of mESCs (Baillie-Johnson et al., 2014; van den Brink et al., 2014), we show that a similar treatment of cell aggregates results in the emergence of a directional extension that is neural in character and harbours, at its distal end, a population that resembles the NMp in terms of gene expression and phenotypic behaviour. We discuss the signalling requirements for the emergence and differentiation of the NMp, and also the role that 3D structures might play in its maintenance.

## RESULTS

### Wnt/β-catenin signalling promotes the emergence of posterior axial fates in adherent mESC differentiation

**Fig. 1.**
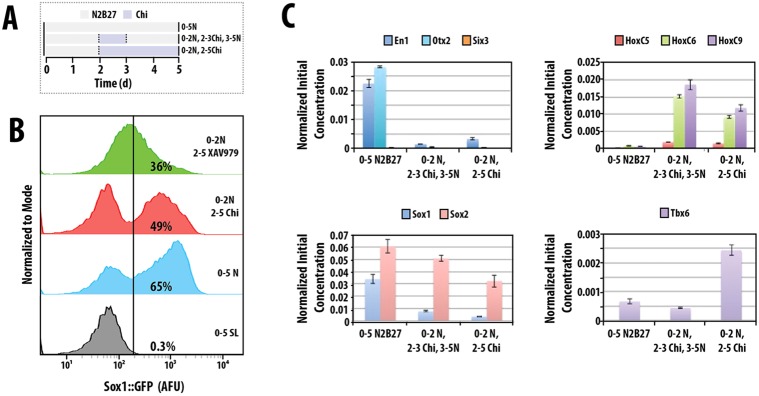
**Wnt signalling is transiently active during neural differentiation and does not inhibit neural development.** (A) The differentiation protocol. (B) Population profiles of Sox1::GFP mESCs treated with continuous Serum+LIF (0-5 SL) or N2B27 (0-5 N) or for 2 days with N2B27 followed by either Chiron (2-5 Chi) or the tankyrase inhibitor XAV939 (2-5 XAV939). Black vertical bisecting line indicates the boundary between Sox1::GFP-negative and -positive, as determined by the autofluorescence profile from wild-type (E14-Tg2A) cells. Population profiles normalized to the mode fluorescent value of the population. (C) mRNA from Sox1::GFP mESCs treated with N2B27 for 5 days, or with N2B27 and a Chiron pulse on days 2-3 or 2-5 were analysed by qRT-PCR for anterior (*En1*, *Otx2*, *Six3*), spinal cord (*Hoxc5*, *Hoxc6*, *Hoxc9*), neural (*Sox1*, *Sox2*) and mesoderm (*Tbx6*) identifiers. Significance tests were performed on this set of experiments and are presented in supplementary material Fig. S2. Data are mean±s.d.

In the postimplantation epiblast of the mouse embryo, cells become allocated to one of two prospective fates: anterior neuroectoderm (aNECT) that will give rise to the anterior nervous system; or, posteriorly, to a rapidly expanding population that gives rise to the mesoderm and the endoderm through the primitive streak (Arnold and Robertson, 2009; Rossant and Tam, 2009). Aspects of this decision-making event can be modelled in ESC cultures where fates are controlled by extracellular signals. Thus, differentiation in the presence of nodal, activin, BMP and Wnt/β-catenin signalling promotes the emergence of a primitive streak-like population (Gadue et al., 2005), whereas culture in N2B27 or serum and retinoic acid (RA) generates neural precursors that can be monitored in the expression of Sox2 and a Sox1::GFP reporter (Ying et al., 2003; Abranches et al., 2009). After 2 days of differentiation in N2B27, mESCs commit either to a neural or to a mesendodermal fate in a signal-dependent manner (Thomson et al., 2011; Turner et al., 2014b). This commitment is associated with a transient rise in Wnt/β-catenin signalling (Faunes et al., 2013) (supplementary material Fig. S1) that can be shown to promote both fates, depending on the levels of nodal and activin: together with high levels it promotes mesendodermal fates; in the context of low levels, it is required for neural differentiation (Turner et al., 2014b), as shown by the effects of inhibition of Wnt signalling (Fig. 1B). This suggests that the activation of Wnt signalling observed during the second day is part of a general priming of the differentiated state and raises the question about its role in the development of the nervous system.

Wnt/β-catenin signalling has been shown to promote the maintenance of neural progenitors and their maturation into neurons (Zechner et al., 2003; Otero et al., 2004; Kim et al., 2009; Slawny and O'Shea, 2011); this could account for the effects of Wnt/β-catenin signalling during neural differentiation. However, Wnt/β-catenin signalling also plays a role in the specification of axial identities in developing embryos (Kiecker and Niehrs, 2001; Nordström et al., 2002, 2006; Mazzoni et al., 2013) and we have tested whether this is also the case during mESC differentiation.

mESCs differentiating in N2B27 adopt a neural fate and, after 5 days, express high levels of genes that are characteristic of mid/hindbrain (*En1*, *Otx2*), but lack expression of spinal cord identifiers (*Hoxc5*, *Hoxc6*, *Hoxc9*) (Fig. 1C). However, exposure to Chiron after 2 days in N2B27 elicits the expression of thoracic Hox genes in the absence of anterior ones (Fig. 1C), indicating that β-catenin promotes the development of posterior fates in mESCs. To test whether this effect required persistent signalling, cells were exposed to Chiron for shorter periods of time. Exposure of mESCs to Chiron between 48 and 72 h of differentiation elicits the emergence of thoracic spinal cord identities at the expense of anterior fates (Fig. 1C). A difference between the continuous and pulsed exposure to Chiron can be observed in the cell types that emerge from this population: in both cases, Chiron suppresses the differentiation of anterior identities but, in the case of continuous exposure, in addition to neural development Chiron promotes mesoderm differentiation, as shown by the expression of *Tbx6* (Fig. 1C). These results are in agreement with the notion that high levels of Wnt signalling lead to the emergence of a cell population with posterior identities in the epiblast (Mazzoni et al., 2013; Tsakiridis et al., 2014) and raises the issue of the mechanism through which this is achieved.

### Wnt/β-catenin signalling promotes the emergence of an axial progenitor population in adherent mESC differentiation

**Fig. 2.**
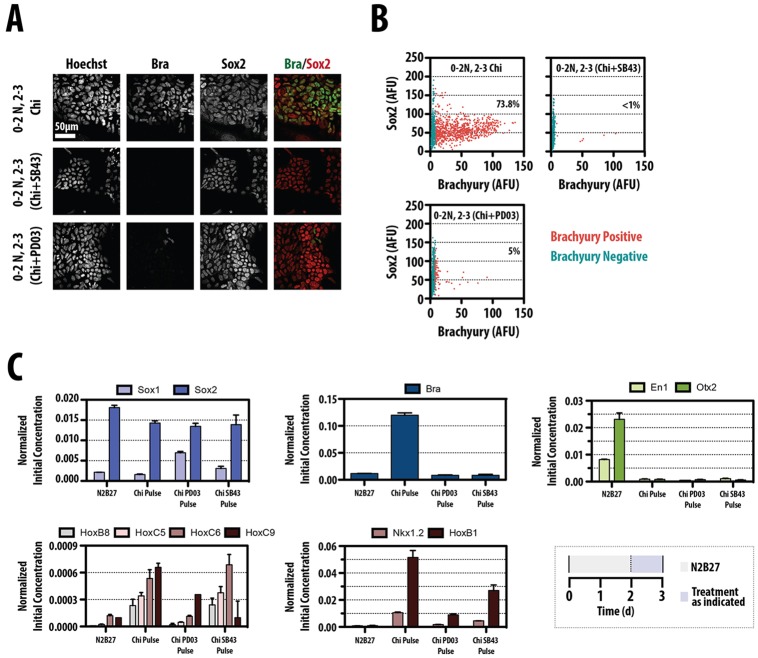
**Wnt/β-catenin signalling promotes the emergence of an NMp precursor in cell culture.** (A) Confocal images of Sox1::GFP mESCs exposed to 2 days N2B27 followed by 24 h Chiron alone (Chi) or in combination with SB43 or PD03 (Chi+SB43 and Chi+PD03, respectively) fixed and immunostained for Bra (green) and Sox2 (red). Hoechst was used to identify the nuclei. Scale bar: 50 µm. AFU, arbitrary fluorescence units. (B) Quantitative image analysis of the confocal images from the conditions in A. Teal blue dots indicate cells with no Bra (95% of cells in either Chi+PD03 or Chi+SB43); red dots correspond to cells with significantly high levels of Bra protein (percentage of Bra-positive cells indicated). (C) Sox1::GFP mESCs were treated as described in A prior to RNA extraction and qRT-PCR analysis for genes associated with neural (*Sox1*, *Sox2*), primitive streak (*Bra*), anterior neural (*En1*, *Otx2*) and anterior/posterior regional (*Hoxb1*, *Hoxc5*, *Hoxc6*, *Hoxc9*) identities, and the NMp (*Nkx1.2*, *Hoxb8*). Data were normalized to the house-keeping gene *Ppia* and are expressed as sample concentrations±s.d. Significance tests were performed on this set of experiments and are presented in supplementary material Fig. S2.

Exposure of mESCs to Chiron between 48 and 72 h elicits the appearance of clusters of cells that co-express Sox2 and Bra (Fig. 2A,B), a signature that is characteristic of a population of cells that have been suggested to act as a precursor of the spinal cord and the paraxial mesoderm in the embryo, the neuromesodermal axial precursor (NMp) (reviewed by Wilson et al., 2009; Kondoh and Takemoto, 2012). Addition of SB43, a TGFβ antagonist, simultaneously with Chiron, abolishes the expression of Bra, and thus of the Sox2^+^ Bra^+^ subpopulation. A similar effect is observed if FGF signalling is inhibited by exposure to PD03, an inhibitor of MEK. This suggests that the population is associated with the specification of mesendodermal fates that have been shown to be the source of this population.

Analysis of gene expression in cells 72 h after exposure to Chiron between 48 and 72 h shows that they express Sox1 and Sox2, as well as markers associated with the NMp: *Bra*, *Nkx1.2* and *Hoxb8* (Delfino-Machin et al., 2005) (Fig. 2C). These cells do not express markers of anterior neural fate, e.g. *En1* and *Otx2*, but exhibit low levels of posterior ones, e.g. *Hoxb1*, *Hoxc5*, *Hoxc6* and *Hoxc9* (Fig. 2C). This signature requires ERK and nodal/activin signalling (Fig. 2), and is dependent on Chiron, as cells differentiating in N2B27 for the same period of time do not express Bra or posterior markers, but do express anterior fates.

**Fig. 3.**
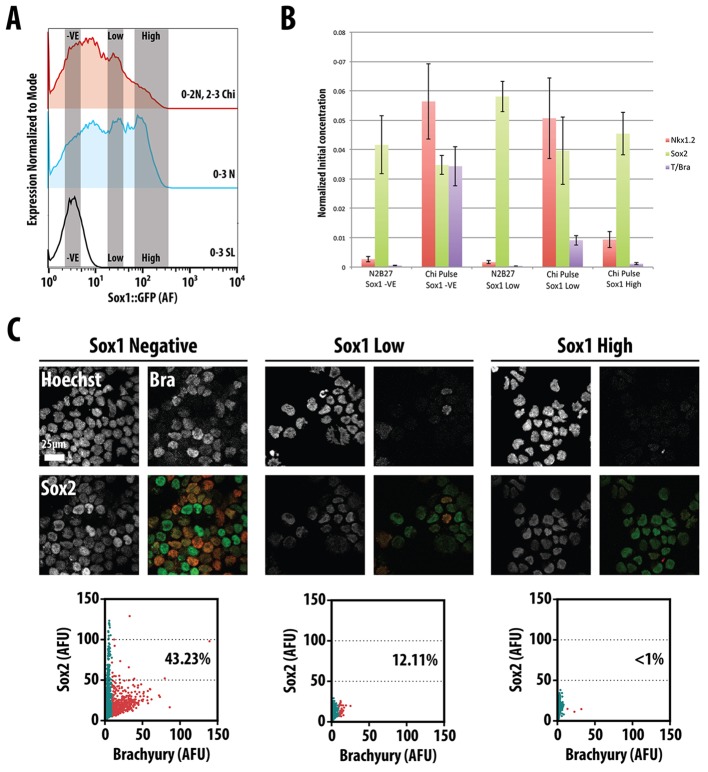
**Identification of the NMp amidst differentiating neural cells on day 3 of differentiation.** (A) Population profiles of Sox1::GFP mESCs analysed by FACS following treatment with a 24 h Chiron pulse after 2 days N2B27 (Chiron pulse, upper panel), 3 days N2B27 (middle panel) or 3 days serum+LIF (0-3 days SL, lower panel). Grey vertical boxes highlight the negative, low and high populations. (B) Cells sorted from the negative, low and high Sox1::GFP populations were analysed by qRT-PCR for the presence of *Nkx1.2* (red), *Sox2* (green) and Brachyury (Bra, purple). Data are mean±s.d. Significance tests were performed on this set of experiments and are presented in supplementary material Fig. S3. (C) The negative, low and high Sox1::GFP populations from the Chiron pulse condition were fixed and stained for Brachyury and Sox2. Quantitative image analysis of the fixed cells shown below each condition. Teal blue dots indicate cells with no Bra (95% of cells in either Chi+PD03 or Chi+SB43), whereas red dots correspond to cells with significantly high levels of Bra protein (percentage of Bra-positive cells indicated). The NMp is found in cells negative or low for Sox1::GFP.

To study the emergence of the Sox2^+^ Bra^+^ Nkx1.2^+^ population in the context of neural differentiation, we analysed gene expression in subpopulations of cells expressing Sox1::GFP, a marker of mature neural progenitors. After 3 days differentiating in N2B27, Sox1::GFP-expressing cells exhibit a complex expression profile with a prominent negative population (Fig. 3A). Addition of Chiron during the third day reduces the Sox1::GFP high population in favour of the negative one (Fig. 3A). To search for the NMp gene expression signature within this profile, we used FACS to sort cells with no, low or high levels of Sox1::GFP, and analysed them for the presence of the gene expression signature of the NMp. The three subpopulations expressed *Sox2*, with lowest levels in the negative population, but could be distinguished by the levels of expression of *Bra* and *Nkx1.2*, which were highest in the negative population, noticeable in the low and absent in the high (Fig. 3B). Immunocytochemical analysis of the three populations reveals differences in the proportion of Sox2^+^ Bra^+^ cells, with the Sox1::GFP negative population having the highest, with all Bra^+^ cells expressing Sox2 (Fig. 3C). These results indicate that exposure of mESCs to Chiron during day 3 of differentiation induces the appearance of a population that has many of the hallmarks of the NMp that has been postulated to emerge during the late stages of gastrulation.

### A window of competence for the emergence of the axial progenitor

**Fig. 4.**
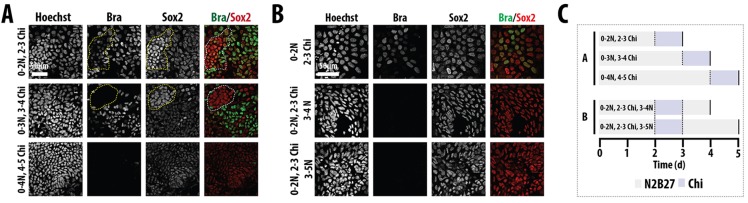
**A window of competence exists for the generation of the NMp.** E14-Tg2A mESCs were exposed to either (A) a 24 h pulse of Chiron after 2, 3 or 4 days in N2B27 (0-2N 2-3Chi, 0-3N 3-4Chi, 0-4N 4-5Chi, respectively) and fixed or (B) a 24 h pulse of Chiron after day 2 and returned to N2B27 for a further 24 or 48 h before fixation and staining for Sox2 and Bra. (C) Schematic representation of the stimulation protocol. The highest proportion of cells that co-express Sox2 and Bra, and therefore the highest proportion of NMp cells, occurs when cells are exposed to a 24 h pulse during days 2-3. Hoechst indicates the nuclei. Scale bars: 50 µm.

To determine whether there is a period of competence for the induction of the Sox2^+^ Bra^+^ population, we altered the timing of the exposure to Chiron (Fig. 4A). Treatment of the differentiating cell population between 72 and 96 h reduces the number of Sox2^+^ Bra^+^ cells and this number is further reduced if the exposure occurs between 96 and 120 h (Fig. 4). When mESCs that had been exposed to Chiron (Fig. 4B) between 48 and 72 h of differentiation are returned to N2B27, the Sox2^+^ Bra^+^ population decays rapidly over the next 2 days, and cannot be found by day 5 (Fig. 4). In both cases, the cells maintain Sox2 expression. These results suggest that there is a sensitive period on the third day of differentiation in which this population can be induced and that, in the absence of signalling, the population is not maintained and adopts a neural fate.

### Single-cell analysis of ESC-derived NMps

**Fig. 5.**
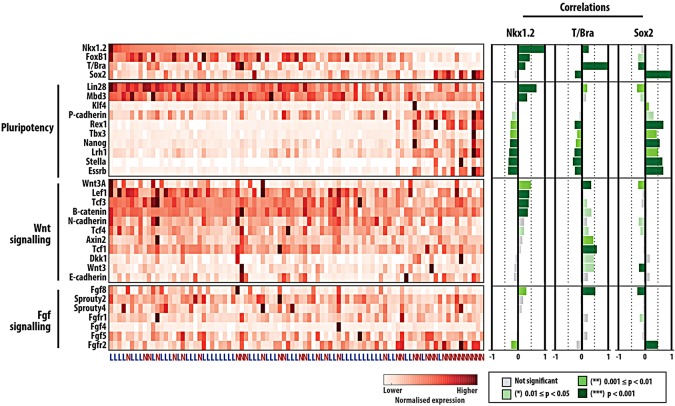
**Single-cell analysis of the NMp population derived from mESCs.** mRNA was extracted from Sox1::GFP single cells expressing either low (L, 53 cells) or negative (N, 36 cells) GFP levels and analysed for the indicated genes using Fluidigm technology. Gene expression levels for individual cells are shown in colour-code and ordered according to the levels of *Nkx1.2* expression (top). On the right, Pearson correlation coefficients of the expression of each given gene to *Nkx1.2*, *Bra* or *Sox2* are displayed and colour coded according to statistical significance level. The expression of pluripotency markers, as well as of Wnt and FGF signalling pathway components in individual cells, is shown here (see supplementary material Figs S4 and S5 for other markers and extended descriptions of data acquisition and processing).

**Fig. 6.**
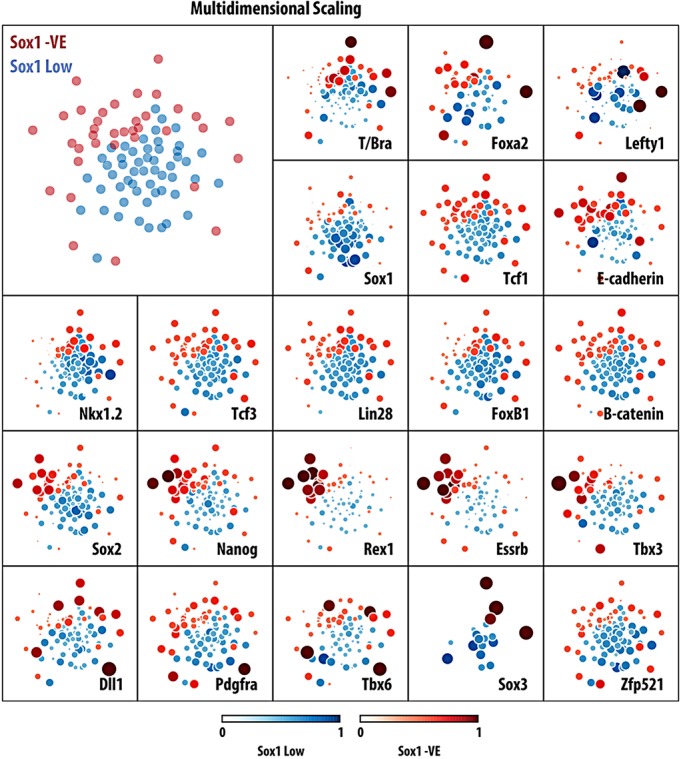
**Dimensionality reduction analysis of single-cell expression data of the NMp population derived from mESCs.** Multidimensional scaling (MDS) was applied to reduce the 85-dimensional single-cell profiles into a two-dimensional dataset and facilitate visual inspection. In contrast to other dimensionality reduction methods, MDS (top left panel) was able to disaggregate, albeit only partially, low and negative Sox1:GFP cell populations (blue and red dots, respectively). Expression levels of some of the assayed genes are shown in colour and size code for each individual cell placed on the corresponding MDS coordinates. The clusters of pluripotent cells within the negative population are markedly highlighted with this data representation.

To gather further information about the presence of NMp-like cells in the negative and low Sox1::GFP populations of mESCs that had been exposed to Chiron, we sorted 192 single cells from both subpopulations (96 negative and 96 low). After a strict quality control, we selected 89 cells (53+36, see supplementary material Fig. S4 for details) and analysed them for expression of 85 genes, including pluripotency markers, lineage markers, axial identifiers and reporters of signalling pathway activity (see Materials and Methods, Fig. 5 and supplementary material Fig. S4). We first tried to order the cells using the conventional NMp signature: *T/Bra*, *Sox2*, *Nkx1.2* in a correlation analysis (Fig. 5, see also supplementary material Figs S4 and S5). Most of the cells analysed have detectable levels of *Nkx1.2*, with the expression of Bra being heterogeneous and slightly correlated with the levels of *Nkx1.2* (correlation coefficient of ∼0.3, *P*<0.001). Surprisingly, the expression of *Sox2* mRNA is low in all cells except for a small group and exhibits no significant correlation with the expression of *Nkx1.2*; this is not a failure to amplify the mRNA as the pluripotent population that can be observed within the Sox1::GFP-negative population exhibits high levels of *Sox2* expression (Fig. 5). A search for genes whose expression is correlated with *Nkx1.2* and *Bra* identified *Wnt3a*, *Fgf8*, *Foxb1*, *Lin28* and *Mdb3* (Fig. 5 and supplementary material Figs S4 and S5). In the embryo, *Fgf8* and *Wnt3a* are expressed in the area in which NMps reside (Delfino-Machin et al., 2005) and thus validate our analysis. *Foxb1* is a member of the Fkh family of transcription factors that has been reported to be expressed in the NMp region during elongation (Ang et al., 1993; Zhao et al., 2007), and thus represents a novel marker for the NMp. However, *Lin28*, a microRNA-binding protein (Viswanathan and Daley, 2010; Shyh-Chang and Daley, 2013), and *Mbd3*, a core member of the NurD chromatin remodelling complex (Kaji et al., 2006; Hu and Wade, 2012), have been associated with pluripotency and reprogramming, and might represent a signature of ‘stemness’. Next, we performed different dimensionality reduction analyses of the data, including principle component analysis (PCA) and linear discriminant analysis (LDA), but these methods were unable to capture structural properties of the cell population, most probably due to the heterogeneous nature of the data. The method that performed best was multidimensional scaling (Fig. 6), which was capable of separating the Sox1::GFP-negative and -low populations. From this analysis we can conclude that the two populations are very similar but that the negative cells contain a subpopulation that has not yet completed a transition from the pluripotent epiblast, as evidenced by a prominent group of cells with high levels of all pluripotency markers, including *Sox2*. The analysis also allows visualization of differences in gene expression between the cells from the two populations (Fig. 6).

The NMp population does not exhibit widespread expression of lineage markers and lacks clear expression of axial markers, except for *Hoxb1*, which is active in the NMp region in the embryo (van de Ven et al., 2011). Interestingly, amidst the analysed population we observed five cells that express *Tbx6*, *Pdgfra* and *Dll1* and are likely to be mesodermal, and 40 cells that express *Sox1*, *N-cadherin* and *Zfp521* and are likely to be neural (supplementary material Fig. S6). These cells are negative for both *Nkx1.2* and *Bra*, and represent progenitors of the mesoderm and the nervous system. These results are consistent with the notion that exposure of mESCs to Chiron during the third day of differentiation elicits the emergence of cells with the signature of the NMp.

### FGF and Wnt signalling are active in NMps

The single-cell analysis reveals expression of targets of FGF (*Spry2*, *Spry4*) and Wnt (e.g. *Lef1*, *Dkk* and *Axin2*) signalling in cells that express *Nkx1.2*, an indication of FGF/ERK and Wnt/β-catenin signalling in this population. This is consistent with results from embryos (Storey et al., 1998; Mathis et al., 2001) and with the requirements for these signalling pathways in the specification of the NMp population (Fig. 2A,C). By contrast, there is no clear indication of BMP or nodal signalling (supplementary material Fig. S4). These observations led us to test the roles of FGF in the specification and differentiation of the NMp population.

**Fig. 7.**
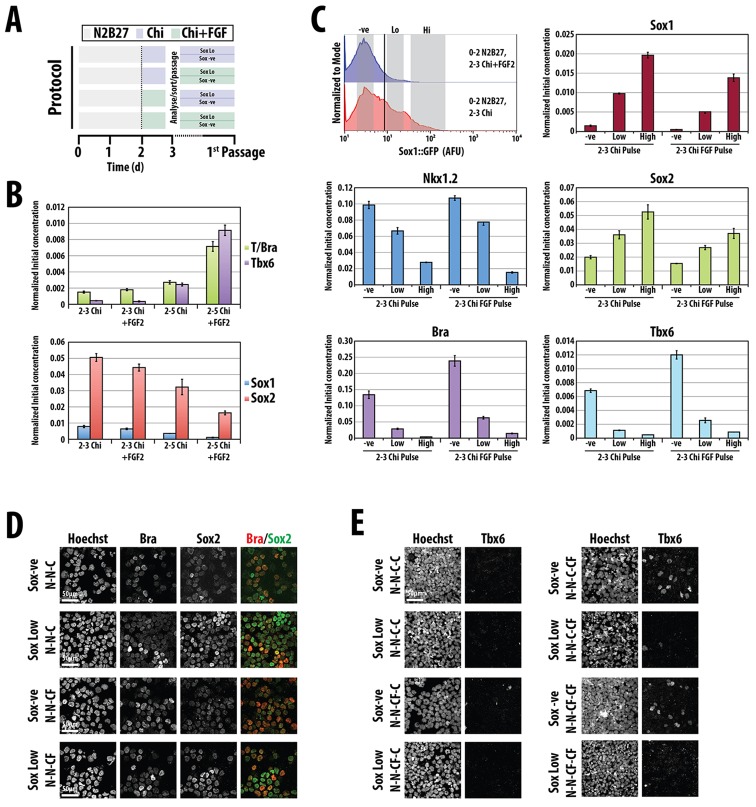
**Role of FGF in NMp establishment and differentiation.** (A) Differentiation protocols used throughout this figure. (B) mRNA from E14-Tg2A mESCs treated with a 24 h pulse of Chiron or Chiron and FGF2 on days 2-3, or with Chiron or Chiron and FGF2 from days 2-5 were analysed by qRT-PCR for *Bra* and *Tbx6* (top) or *Sox1* and *Sox2* (bottom). Data presented as mean±s.d. Significance tests were performed on this set of experiments and are presented in supplementary material Fig. S2. (C) Population profiles of Sox1::GFP mESCs differentiated after 5 days incubation with N2B27 with a 24 h pulse between days 2 and 3 of either Chiron and FGF or Chiron. Grey boxes indicate the negative, low- and high-expressing populations. mRNA from cells that were sorted from the indicated populations and conditions was analysed for the indicated genes by qRT-PCR. Data presented as mean±s.d. Significance tests were performed on this set of experiments and are presented in supplementary material Fig. S3. (D) Sox1::GFP mESCs exposed to Chi on days 2-3 were sorted based on the level of expression (negative or low) and stained for Sox2 and Bra. (E) Tbx6 expression from sorted Sox1::GFP mESCs after a first passage under the indicated conditions. N, N2B27; C, Chiron; F, FGF. N-N-C means 2 days in N2B27 and 1 day in Chiron, etc.

Exposure to both Chiron and FGF between 48 and 72 h leads to a strong suppression of *Sox1*, a decrease in *Sox2* levels and an increase in *Bra* expression relative to Chiron alone (Fig. 7B,C). This results in an increased number of NMps after exposure to these signals and suggests that, in combination with Wnt/β-catenin signalling, FGF promotes early progenitors at the expense of differentiating cells that are likely to become mesoderm. During differentiation, continuous exposure to both FGF and Chiron between 48 and 120 h amplifies the emergence of Wnt/β-catenin-dependent mesodermal derivatives, as reflected in *Tbx6* expression (Fig. 7). To determine whether this is due to an effect on the NMps, Sox1::GFP-negative and -low cells were sorted from pulsed exposure to either Chiron or Chiron and FGF, and each fraction was further cultured in either Chiron or Chiron and FGF. After one and two passages, cells were tested for Sox1, Sox2 and Tbx6 expression. Only the Sox1::GFP originally negative population gave rise to Tbx6-expressing cells and the number of Tbx6-positive cells was enhanced in the presence of FGF (Fig. 7E). This analysis suggests that, in culture, cells within the NMp-like population are in a state that reflects the situation in the embryo in terms of signalling requirements, particularly FGF and Wnt, and can differentiate into both neural and paraxial mesodermal precursors in a signal-dependent manner.

### Emergence of an axial progenitor and polarized growth in 3D aggregates of mESCs

**Fig. 8.**
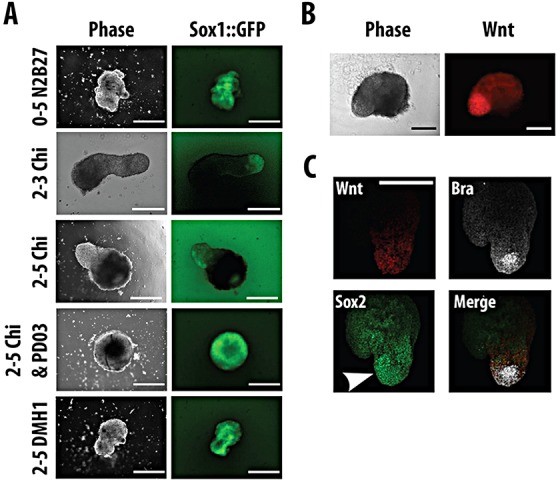
**Elongation process.** (A) Examples of aggregates from Sox1::GFP mESCs aggregated for 2 days in N2B27 before further treatment with N2B27, which may include a 24 h pulse on day 3 with Chiron, 3 days of Chiron, or Chiron and the MEK inhibitor PD03, or 3 days with the BMP inhibitor DMH1. Aggregates were imaged on day 5 by wide-field epifluorescence microscopy. The phase-contrast and fluorescence images shown are representative examples. Aggregates exposed to a 24 h pulse of Chiron are able to show a single large extension containing Sox1::GFP-expressing cells with a region at the tip that is negative for the fluorescence reporter. (B) Wnt reporter TLC2 mESC aggregates differentiated in N2B27 with a 24 h pulse of Chiron on day 3 and imaged on day 5. (C) Aggregate as in B fixed and immunostained for brachyury (white) and Sox2 (green). The fluorescent reporter is expressed predominately in the aggregate extension corresponding to a Sox2^+^ Bra^+^ region. The white arrowhead indicates the region of highest Sox2 expression. Hoechst was used to label the nuclei. Scale bars: 500 µm in A; 200 µm in B,C.

Recently, we have developed a 3D mESC culture system that mimics some of the morphogenetic events taking place during early embryogenesis (Baillie-Johnson et al., 2014; van den Brink et al., 2014). Using Sox1::GFP mESCs and exposing them to continuous differentiation in N2B27, we observe the emergence of Sox1::GFP-positive cells throughout the aggregate (Fig. 8A) and a similar pattern is observed if, on day 3, the aggregates are exposed to DMH1, a BMP inhibitor (Fig. 8A; 2-5 DMH1), SB43, an activin/nodal inhibitor (data not shown) or PD03 (Fig. 8A; 2-5 Chi+PD03). The latter results are in agreement with previous observations that differentiation of embryoid bodies in the absence of external signals leads to the development of neural tissue (Watanabe et al., 2005; Wataya et al., 2008).

**Fig. 9.**
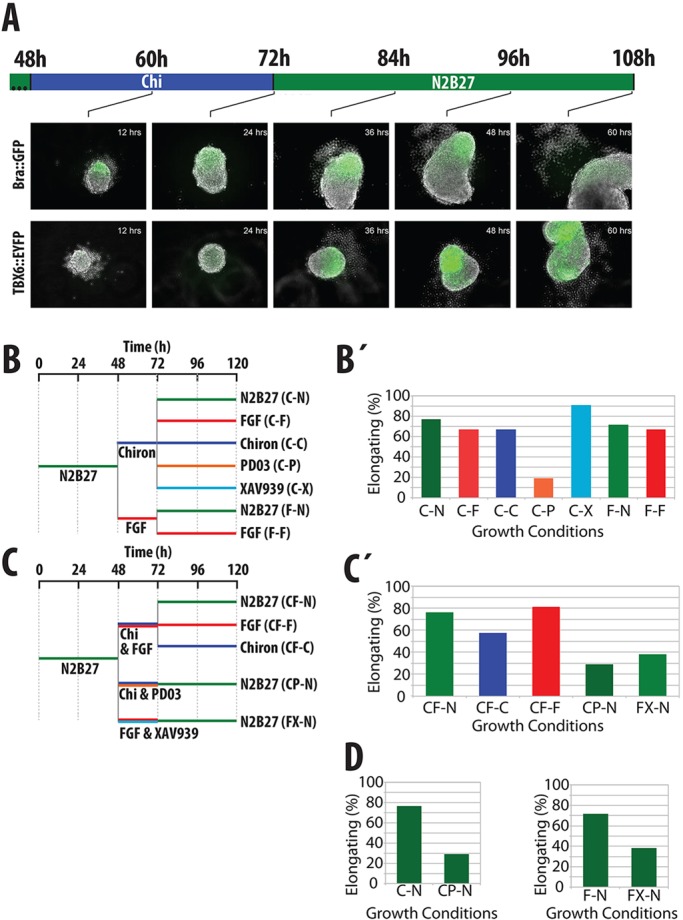
**Signalling requirements in aggregates.** (A) Stills from movies of aggregates from Bra::GFP (top) or TBX6::EYFP mESCs, started at the time of exposure to Chiron (after 2 days in N2B27). Expression of Tbx6 is delayed relative to Bra. Notice the extrusion of cells from the Bra expression domain as well as the elongation. (B,C) Summary of treatments applied to mESC aggregates to test signalling requirements for the maintenance of the elongation (B) and the establishment of the precursor population (C). In all conditions, mESCs are formed as aggregates for 2 days in N2B27 (green) before the medium is changed to include a 24 h pulse as indicated. Growth conditions are abbreviated within the parentheses. (B′,C′) Proportion of aggregates displaying elongated phenotypes from each growth condition. FGF signalling is essential for the maintenance and establishment of the elongation, whereas Wnt signalling is required for the establishment only. (D) Specific comparisons demonstrate the requirements for Wnt and FGF signalling in establishing the precursor population. Abbreviations as in B. Significance tests were performed on this set of experiments and are presented in supplementary material Fig. S7.

When the aggregates are cultured in conditions that elicit the NMp-like population in adherent culture, they exhibit a polarized expression of Bra and undergo a polarized elongation that, after 2 further days in N2B27, has become 200-300 µm long and 20-40 µm wide (Figs 8 and 9). The elongated region expresses Sox1::GFP and Sox2, while the tip exhibits high levels of Bra and of Wnt signalling (Fig. 8). Expression of Bra is confined to the population at the tip, indicating that the elongation lacks a notochord (Fig. 8C). This configuration is very reminiscent of the situation in the caudal lateral epiblast of the developing embryo, where the NMp population is thought to reside between stages 7.5 and 11.0 (Wilson et al., 2009). To test for the presence of paraxial mesoderm in these aggregates, we used mESC carrying a YFP reporter for Tbx6 (van den Brink et al., 2014). As the aggregate begins to elongate, after it has expressed *Bra* and *Sox1* we observe mesenchymal-like cells that express Tbx6::EYFP in the elongating region (Fig. 9A). Most of these cells leave the aggregate and either float in the medium or attach, preferentially, to the opposite end to the elongating tissue; only cells that attach to the aggregate maintain expression of the reporter (Fig. 9). These results suggest that, when elicited in three dimensions, the NMps can organize themselves into structures that resemble the initial steps of spinal cord development and can be maintained autonomously for a number of days.

### FGF regulates the extension of the aggregates

We also tested the role of FGF in the organization and development of the elongates (Fig. 9). Suppression of MEK signalling from 72 to 120 h prevented the growth of the elongations (Fig. 9B′), indicating that it is necessary to support the growth once it has started. Addition of FGF during this period did not have a significant effect, indicating that the endogenous levels produced by the aggregates are sufficient. This effect was also seen for Wnt/FGF inhibition during the 48-72 h period (Fig. 9D), consistent with our observations from adherent culture that FGF signalling is necessary for the establishment of the elongation. Blocking Wnt/β-catenin signalling also affects the elongation, consistent with the effect of mutants in Wnt signalling on axial development in the embryo (Aulehla et al., 2008; Dunty et al., 2008; Aulehla and Pourquie, 2010).

## DISCUSSION

We have shown that mESCs differentiated in adherent culture under the influence of Wnt/β-catenin signalling give rise to cells with the signature and potential of the axial NMps that have been described in the embryo as giving rise to both paraxial mesoderm and neural progenitors. The impact of Wnt/β-catenin signalling in the emergence of this population is most effective on day 3 of differentiation, when mESCs have been shown to transit through a stage that is similar to that of the post-implantation epiblast (Sterneckert et al., 2010; Turner et al., 2014b). This adds support to a recent report that EpiSCs exposed to Wnt signalling develop primitive streak fates, as well as a small subpopulation with the characteristics of the NMp (Tsakiridis et al., 2014).

The NMp-like population that emerges in adherent culture cannot be expanded efficiently under our culture conditions and tends to differentiate into neural precursors and paraxial mesoderm. Applying the same conditions to a 3D system of mESC aggregates that we have developed to study the early stages of embryogenesis, we observed the emergence of a phenotypically similar population that now is maintained for at least 3 days, and drives the polarized elongation of a structure containing, principally, neural progenitors; the maintenance and elongation of this population is driven by FGF signalling. These observations support the suggestion that the spinal cord is derived from an axial progenitor population and provide an experimental system, the aggregates of mESCs, to identify and dissect the networks that establish, maintain and differentiate this population (see also the accompanying paper in this issue: van den Brink et al., 2014). This system is complementary to those that already exist that can generate forebrain and retina (Eiraku et al., 2008, 2011; Sasai et al., 2012; Lancaster et al., 2013; Sasai, 2013), and it should allow the study of the mechanisms that pattern axial derivatives, in particular the nervous system (Sasai et al., 2012).

### Origin of the NMp population

An important unresolved issue in developmental biology relates to the embryological origin of the spinal cord in amniotes, whether its progenitor population emerges from a posteriorization of an anteriorly fated neural plate or as a separate population, induced *de novo* in the primitive streak, which does not go through an anterior fate (Stern et al., 2006; Wills et al., 2010). The first notion is derived from classical experiments in frogs (Nieuwkoop et al., 1952) and finds support from modern work with *Xenopus* and zebrafish. However, in amniotes, the origin of the spinal cord anlage is spatially and temporally separate from that of the fore-, mid- and hindbrain. For example, recent experiments can generate this population directly from EpiSCs, which do not have a neural fate (Tsakiridis et al., 2014), and experiments in chicken and mice provide evidence for the existence of a distinct progenitor population, adjacent to but distinct from the anterior neural plate, that can give rise to neural tissue of different axial levels through long term growth (Brown and Storey, 2000; Mathis and Nicolas, 2000). Although it cannot be ruled out that the NMp population emerges from a group of cells that has an underlying anterior neural fate, these results suggest that posterior fates might arise over time and within cells that do not exist at the time of the initial specification of the population, i.e. the mechanisms for the specification of posterior neural fates might differ between amniotes and anamniotes.

The NMp population emerges within the mouse node-streak border (NSB) at the end of gastrulation and careful lineage-tracing experiments have identified a subpopulation in the regressing node that acts as a source of progenitors, at least for the ventral nervous system (Cambray and Wilson, 2002, 2007). However, removal of the node in chicken and mouse embryos does not affect axial elongation. Genetic or mechanical ablation of the node in mouse embryos before it regresses results in the loss of the notochord and the floor plate, but it does not impair the emergence of a neural tube with spinal cord characteristics, though it is smaller and lacks motor neurons; the paraxial mesoderm is also affected in these embryos (Ang and Rossant, 1994; Weinstein et al., 1994; Davidson et al., 1999; Klingensmith et al., 1999). This observation suggests the existence of alternative sources of progenitors for the spinal cord that are located laterally to the NSB. The elongations that we observe in our aggregates support this possibility as they lack a notochord.

In our experiments, we have observed that signalling on the third day of differentiation is crucial for the commitment of cells to particular lineages both in adherent and 3D culture. At this time, differentiating mESCs transit through an epiblast-like state and therefore our results parallel recent ones showing that transient activation of Wnt signalling in EpiSCs can generate various primitive streak-related populations (Tsakiridis et al., 2014). Altogether, these observations favour the possibility that, at least in culture, an NMp-like population can be generated from a primitive streak-primed epiblast-like population without the need for an anteriorly fated neural template. This is supported by the observation that differentiation of mESCs continuously exposed to activin and Chiron, which suppresses the neural fate, results in the appearance of some Sox2^+^ Bra^+^ cells (Fig. 1A).

### Signalling and the specification and differentiation of the NMp population

In both adherent cultures and aggregates, the appearance of the Sox2^+^ Bra^+^ population is associated with Wnt/β-catenin, as well as with FGF/ERK signalling. Once the population has been established, it appears to use cell-autonomous gene regulatory networks fuelled, as in the embryo, by FGF/ERK signalling for its maintenance and differentiation. Consistent with this, blockage of FGF/ERK signalling after the establishment of the primordium, inhibits its elongation, although it does not affect neural cells generated prior to the inhibition. The details of the structure of this population remain to be elucidated. Retrospective clonal analysis in embryos has suggested the presence of a small, stem cell-like population, with dual potential (Tzouanacou et al., 2009), but this remains to be confirmed. Preliminary results of clonal analysis of the NMp-like population generated in adherent culture have been obtained (P.C.H. and D.A.T., unpublished).

Single-cell analysis of a population enriched for NMps reveals a number of features that will require further examination. Most strikingly, we observe a variation in the levels of *Bra* mRNA expression in the population and a generally low level of *Sox2* expression, which FGF makes very low. These heterogeneities contrast with the more homogeneous and correlated expression of the corresponding proteins. The discrepancy raises the possibility of dynamic transcription time averaging at the level of the population in some of the identifiers of the NMp. Such a situation has been described before for some elements of the pluripotency network (Munoz Descalzo et al., 2013) and might represent a general feature of stem/progenitor cell populations (Martinez Arias and Brickman, 2011). The single-cell analysis also reveals the expression of one new gene in the NMp: *FoxB1*. This gene had been associated with the development of the diencephalon but lineage tracing and gene expression place it within the NMp population (Zhao et al., 2007). Finally, this analysis confirms that the population exhibits high FGF and Wnt signalling, and identifies two genes associated with pluripotency, *Lin28* and *Mbd3*, expressed in these cells, suggesting that they might share some features with mESCs.

In the future, it will be important to explore the properties and long-term renewal and differentiation potential of the NMp-like cells that we have generated in culture and in the 3D aggregates, and compare them with those that have been recently obtained from EpiSCs (Tsakiridis et al., 2014). In this regard, we notice that, under the same conditions, the NMps in the aggregates are maintained over a few days in a manner that they are not in adherent culture. This is likely to be due to the ability of the cells in the aggregate to generate their own niche. It will be interesting to investigate the differences between the two systems to identify the factors that create these differences.

### Note added in proof

While this paper was being reviewed Gouti et al. (2014) have reported a population of cells similar to the one described here during the differentiation of both mouse and human ES cells.

## MATERIALS AND METHODS

### Routine monolayer cell culture, differentiation and aggregate formation

E14-Tg2A, TCF/LEF::mCherry (TLC2), Sox1::GFP, TBX6::EYFP and Bra::GFP mESCs were grown on tissue-culture plastic dishes as described previously (Faunes et al., 2013; Turner et al., 2014b). For differentiation experiments, cells were plated at a density of 4×10^3^ cells/cm^2^ in a base medium of N2B27 (NDiff 227, StemCells) supplemented with combinations of activin A (100 ng/ml), CHIR99021 (Chiron; 3 μM), XAV939 (1 μM), SB431542 (SB43; 10 μM), BMP4 (1 ng/ml), FGF2 (2.5 ng/ml), PD0325901 (PD03; 1 μM) and dorsomorphin-H1 (DM; 0.5 μM). Differentiation medium was replaced daily to reduce the influence of increased concentrations of secreted factors.

To generate aggregates, 40 μl droplets of a 1×10^4^ cells/ml solution (in N2B27) were pipetted into each well of a sterile, non-tissue-culture treated, bacterial grade, U-bottomed 96-well plate with a multichannel pipette. After 48 h, 150 μl of differentiation medium was added directly to the cells and 150 μl was replaced daily. It was essential that during medium replacements, cells were agitated by the forceful ejection of medium to prevent their adhering to the plate surface; medium was not changed during the initial 48 h of aggregate formation. For a full protocol and troubleshooting, see Baillie-Johnson et al. (2014).

### Flow cytometry and microscopy

Cells were analysed for GFP fluorescence using an LSR Fortessa (BD Bioscience) with a 488 nm laser and emission was measured using a 530/30 filter. Forward- and side-scatter properties alongside DAPI exclusion (405 nm laser and emission at 450/50) were used to select live, single cells for analysis in FlowJo (TreeStar). Data were analysed using FlowJo software. Sox1::GFP mESCs were sorted according to their GFP fluorescence in a MoFlo sorter (Beckman Coulter) using the same laser and filter sets described above. Immunofluorescence was performed as described previously (Turner et al., 2014a); a list of primary antibodies and staining conditions, as well as details of data capture are also fully described previously (Turner et al., 2014a).

Epifluorescence, time-lapse imaging of adherent cells was performed with an AxioObserver inverted microscope (Carl Zeiss) in a humidified CO_2_ incubator (37°C, 5% CO_2_). Images were captured every 10 min for the required duration using a 20× LD Plan-Neofluar 0.4 NA Ph2 objective with correction collar adjusted for imaging through plastic. All media were changed daily. An LED white-light system (Laser2000, Kettering, UK) provided illumination. The filter cubes GFP-1828A-ZHE (Semrock) and Filter Set 45 (Carl Zeiss) were used for GFP and RFP, respectively. Emitted light was recorded using an AxioCam MRm and recorded with Axiovision release 4.8.2. For live imaging of cell aggregates, aggregates within a 35 mm non-tissue-culture grade bacterial dish were imaged every 10 min within a humidified (37°C, 5% CO_2_) Biostation IM (Nikon). Analysis performed using Fiji (Schindelin et al., 2012).

### Quantitative RT-PCR

Protocols used are as described previously (Faunes et al., 2013) (see supplementary material Figs S2 and S3). The primers used are available in supplementary material Table S2. The population qRT-PCR experiments are data from one experiment (each sample analysed in triplicate) and are reflective of two independent experiments, whereas, due to constraints of sample availability, the sorted subpopulation qRT-PCR data are from one experiment with each sample analysed in duplicate or triplicate where possible. The Sox1::GFP population profiles gained in each condition have been analysed on multiple occasions and are both reproducible and stable.

### Fluidigm

A list of primers and details of the protocols followed for the Fluidigm analysis have been described previously (Turner et al., 2014b) and are available in supplementary material Table S3 and supplementary materials and methods.

## Supplementary Material

Supplementary Material
