# Supplementary material for: Wnt/β-catenin and FGF signalling direct the specification and maintenance of a neuromesodermal axial progenitor in ensembles of mouse embryonic stem cells
Source: Development. 2014 Nov 15;141(22):4243–53. doi: 10.1242/dev.112979 (PMC4302903; doi:10.1242/dev.112979)
Supplement: Supplementary Material [file supp_141_22_4243__index.html]

Supplementary Material 

# Wnt/β-catenin and FGF signalling direct the specification and maintenance of a neuromesodermal axial progenitor in ensembles of mouse embryonic stem cells

## DEV112979 Supplementary Material

**Files in this Data Supplement:**

- Supplementary Material
