# Supplementary material for: Wnt/β-catenin and FGF signalling direct the specification and maintenance of a neuromesodermal axial progenitor in ensembles of mouse embryonic stem cells
Source: Development. 2014 Nov 15;141(22):4243–53. doi: 10.1242/dev.112979 (PMC4302903; doi:10.1242/dev.112979)
Supplement: Supplementary Material [file supp_141.22.4243_DEV112979supp.pdf]

Supplementary Figure legends

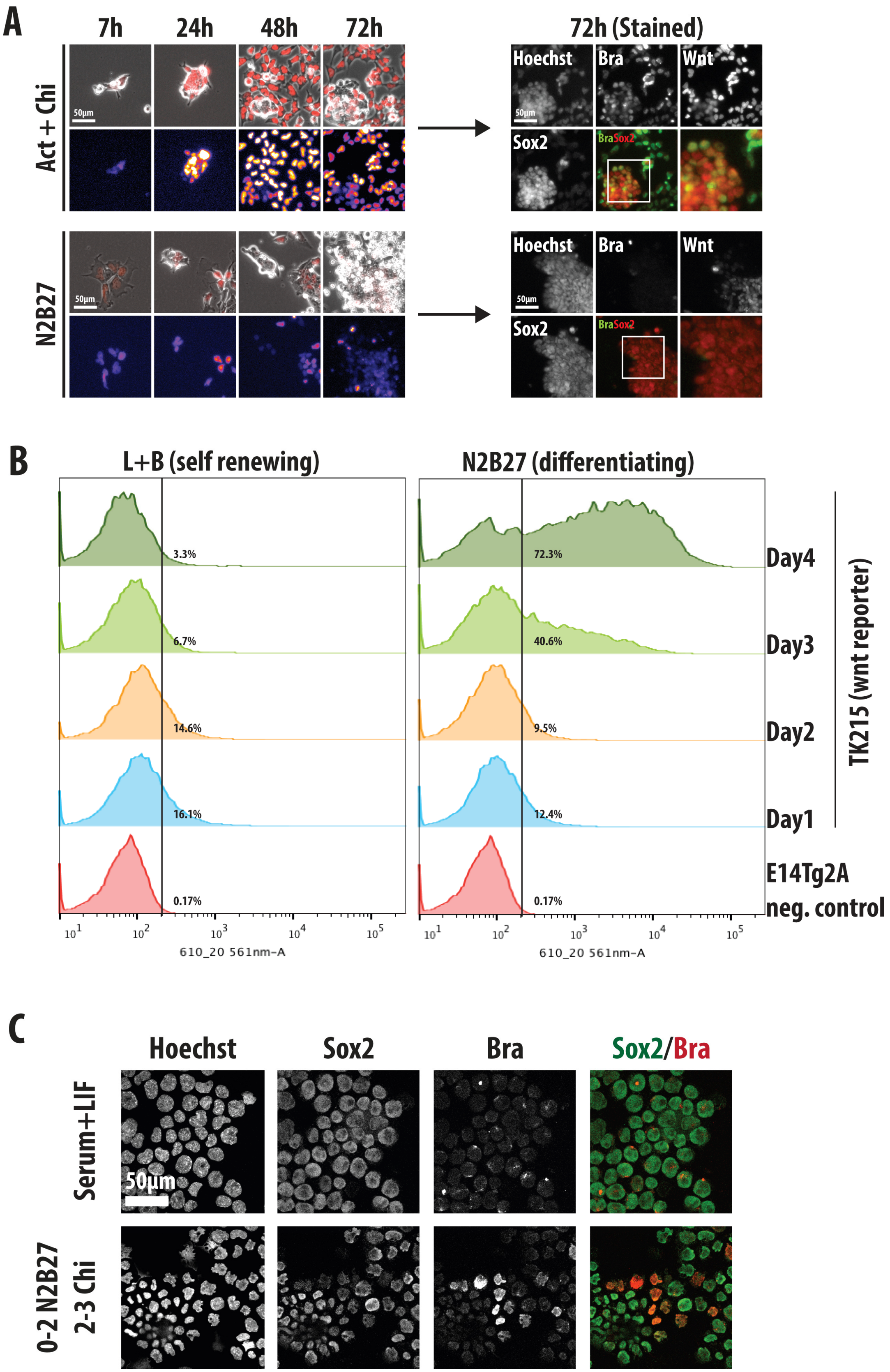

S1. **Endogenous Wnt signalling activity is activated during differentiation in N2B27. (A)** The β-catenin transcriptional reporter, TCF/LEF-mCherry (TLC2), differentiated towards either a Primitive Streak-like (ActivinA+Chiron, top) or neural fate (N2B27, bottom) and imaged by wide-field live-cell microscopy for 72 hours before fixation and immunostaining for Brachyury (Bra; green) and Sox2 (red). White box in each panel represents the magnified region. Scale bars indicate 50µm; Nuclei were labelled with Hoechst. (B) TK215 Wnt signalling reporter mESCs which express mCherry under the control of a Wnt responsive element were plated into either N2B27 (differentiating conditions) or N2B27 containing Lif and BMP (L+B, self-renewing conditions), media was changed daily. The level of Wnt signalling in the cells was assessed by flow cytometry after 24 (days 0-1), 48 (days 0-2), 72 (days 0-3) and 96 hours (days 0-4). (C) Immunostaining for Brachyury (Bra; red) and Sox2 (green) immediately before differentiation (i.e. Serum and LIF) and after differentiation (0-2 N2B27, 2-3 Chi). Scale bar represents 50 µm.

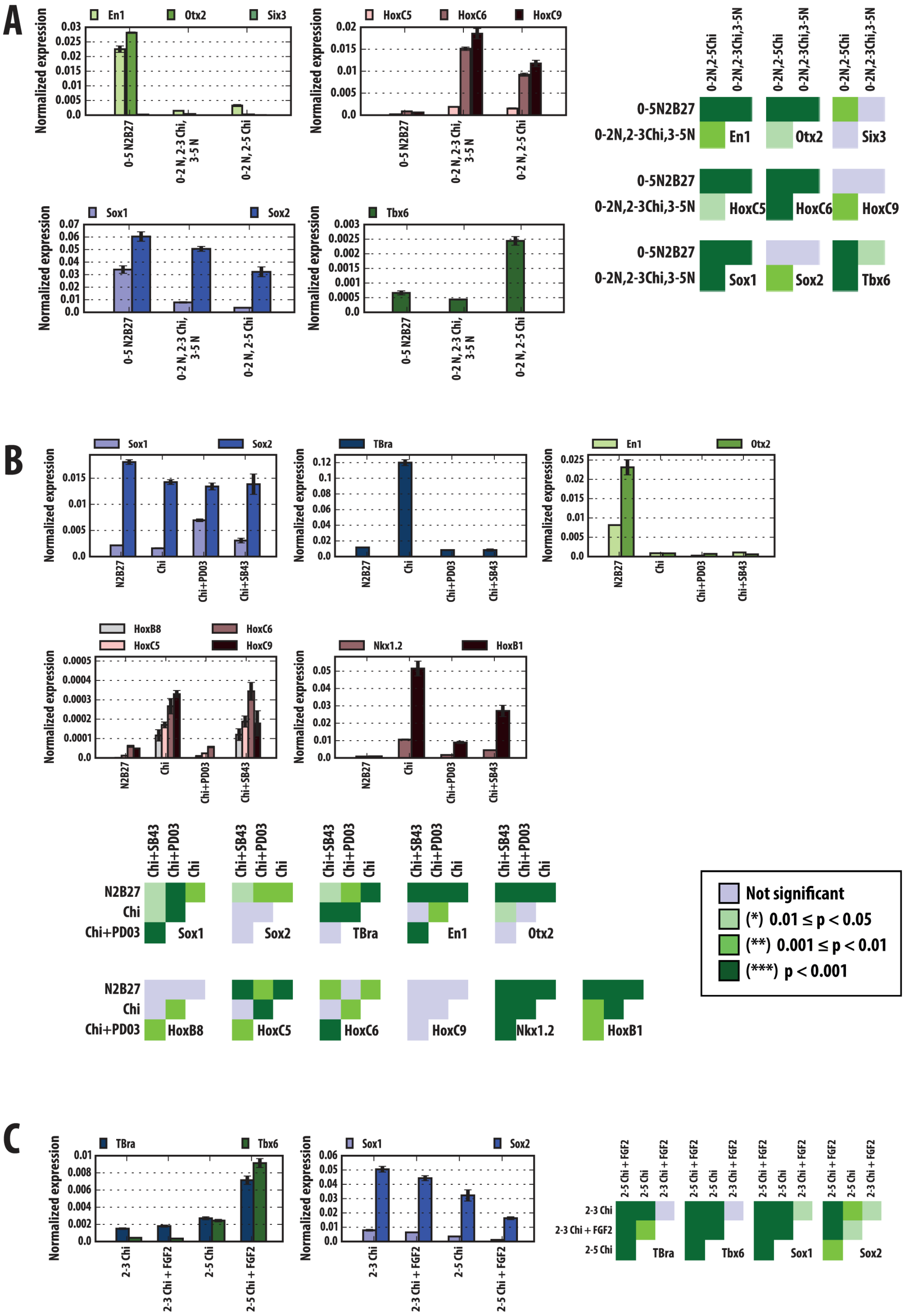

S2. Statistical analysis of gene expression in cell populations with qRT-PCR. mRNA expression levels in cell populations were measured by standard qRT-PCR and quantified

using an in-house implementation of the MAK2 method [Boggy GJ, Woolf PJ (2010). PLoS ONE 5(8): e12355]. Parametric and non-parametric (Mann-Whitney's U and Student's t) tests were performed in order to assess the statistical significance of the differences in mRNA expression levels between different conditions for the assayed genes. To ensure a low type I error we considered that the expression levels of a gene were significantly different between two conditions if they tested positive for both the non-parametric test (α=0.05) and for the parametric test (α_{***}=0.001, α_{**}=0.01, α_{*}=0.05). (A) Sox1::GFP mESCs treated with N2B27 for 5 days, N2B27 with a pulse of Chi on days 2-3 or 2-5 were assayed for anterior (EN1, Otx2, Six3), spinal cord (HoxC5, HoxC6, HoxC9), neural (Sox1, Sox2) and mesoderm (Tbx6 (B) identifiers, see Fig. 1C. (B) Sox1::GFP mESCs cultured in N2B27 for two days, followed by 24h Chiron alone (Chi) or in combination with SB43 or PD03 (C+SB43 and C+PD03 respectively), see Fig. 2C. (C) E24-Tg2A mESCs treated with a 24h pulse of Chi or Chi and FGF2 on days 2-3, or Chi or Chi and FGF2 from days 2-5 were analysed by qRT-PCR for Bra, Tbx6, Sox1 and Sox2, see Fig. 7B.

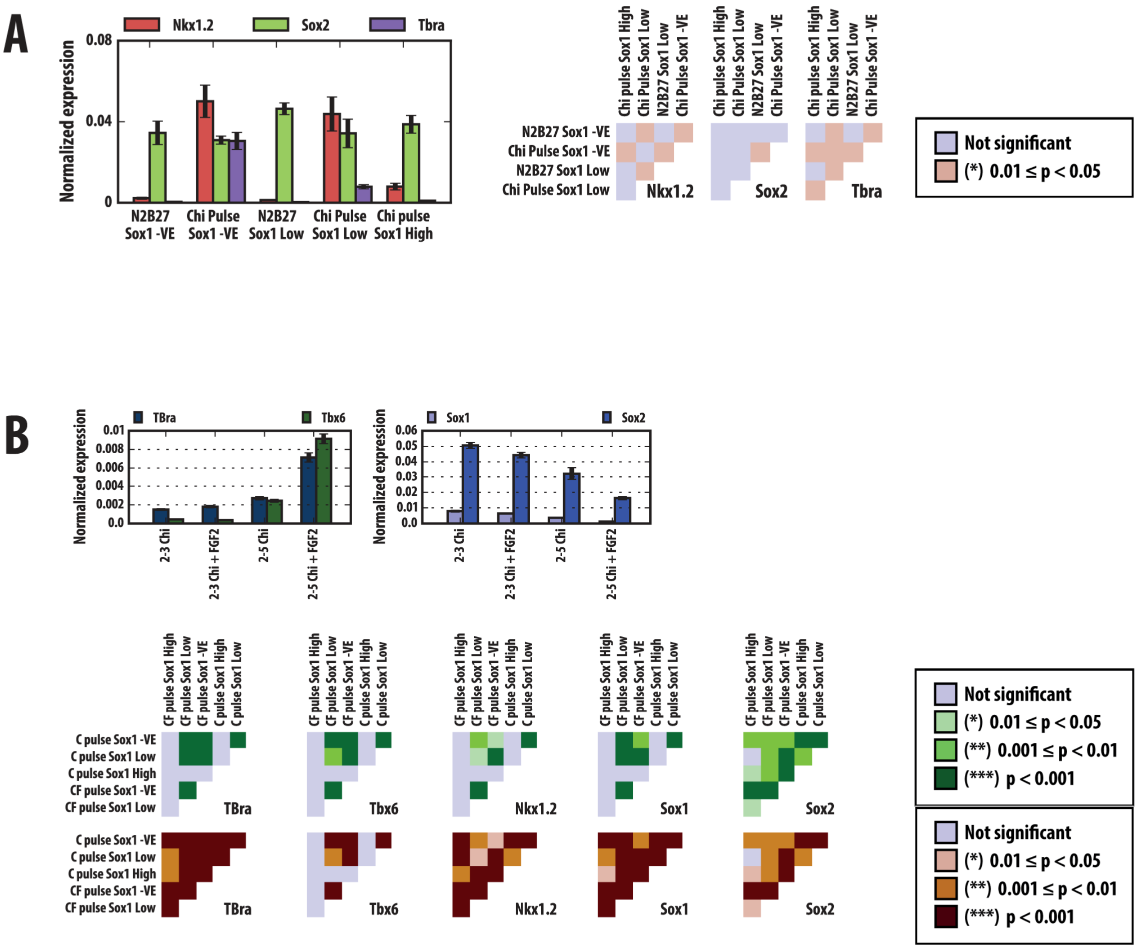

S3. Statistical analysis of gene expression in sorted cell subpopulations with qRT-PCR. (A) Sox1::GFP mESCs were analysed by FACS and sorted into the negative, low and high GFP populations (see Fig. 3B). Expression of Nkx1.2, Brachyury (Bra) and Sox2 was analyzed by qRT-PCR and statistical significance assessed by standard Student's t test (orange-maroon colour scale). (B) Sox1::GFP mESCs differentiated during 5 days in N2B27 with a 24h pulse between days 2 and 3 of either Chiron and FGF or Chiron (see Fig. 7C) were analysed by FACS and sorted into the negative, low and high GFP populations. Expression of Nkx1.2, Brachyury (Bra) and Sox2 was analyzed by qRT-PCR and statistical significance assessed either by a double statistical test as in Fig. S2 (green colour scale of significance). To overcome the lack of statistical significance we also included the significance obtained using standard Student's *t*-tests (orange-maroon colour scale).

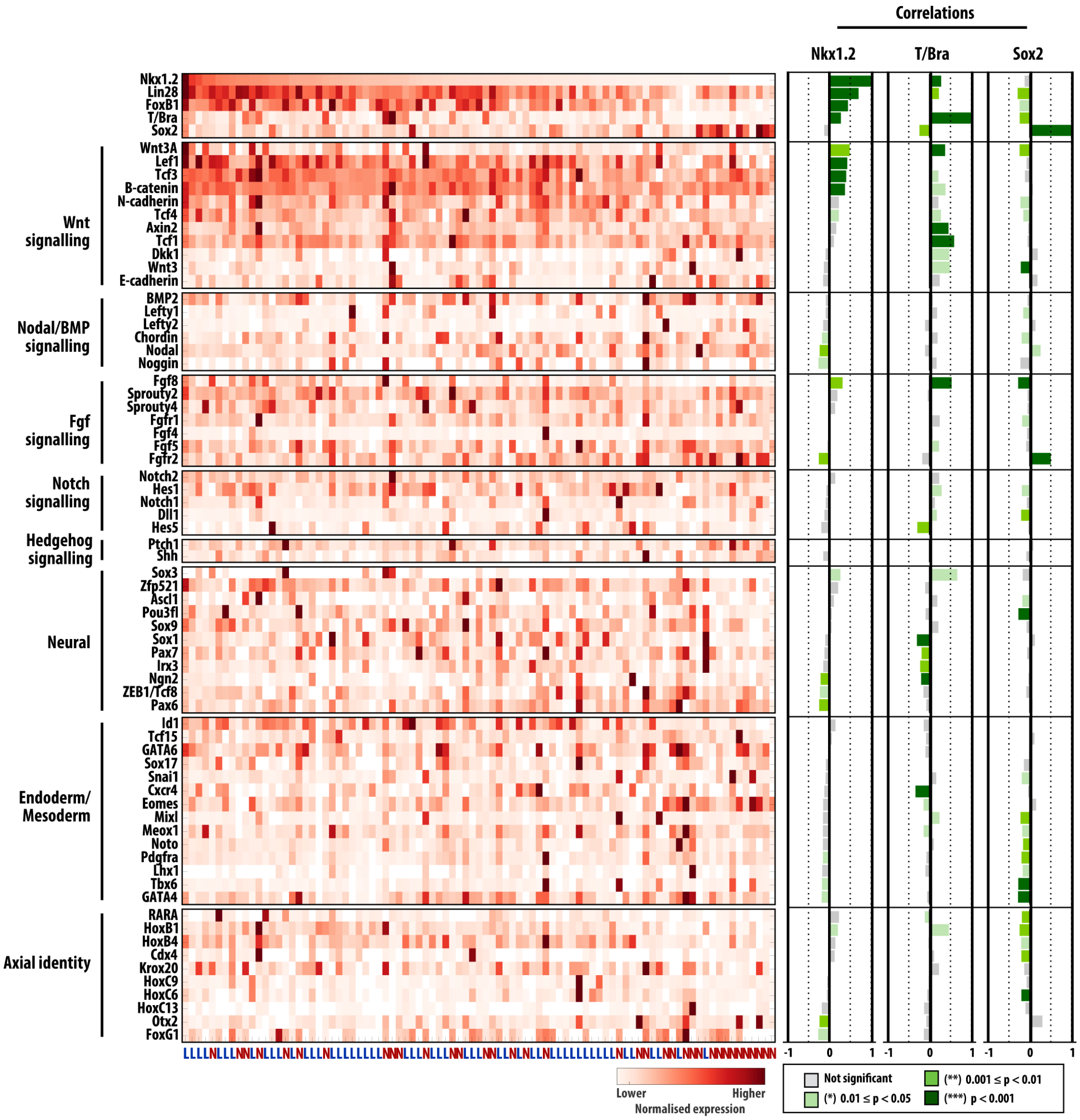

S4. Single cell analysis of the NMp population derived from mESCs. mRNA for 95 genes was extracted from 192 individual Sox1::GFP cells (96 negative for and 96 low) and measured (C_t values) using the Fluidigm platform. We performed a strict quality control on the raw data. Amongst the 18,240 reactions assayed 9,453 (52%) had detectable and reliable expression levels. We removed eight genes which had either amplification of non-specific product or undetectable expression levels in more than 90% of the cells (Emx1, En1, HoxB8, Mesp1, Msgn1, Oct4, Six3 and ZEB2/Sip1). We also removed those cells for which we could not detect levels in at least 10% of the genes (25 and 50 resp.) and those cells for which we failed to detect at least two out of the five control genes (Kdm1a, Mff, Ndufa9, Sdha and Ppia) or with outlying levels in these (18 and 10 cells resp.). We therefore included 86 genes and 89 cells in the analysis (53 Sox1::GFP low and 36 negative). Expression levels normalized to Ppia were computed from Ct values (delta Ct method). In the panel, per gene min-max normalized expression levels for individual cells are shown in colour code and ordered according to the levels of Nkx1.2 expression (top). On the right panel, Pearson correlation coefficient of the expression of each given gene to Nkx1.2, Brachyury (Bra) and Sox1 is displayed. Correlation coefficients shown in colours tested significant (α_{***}=0.001,

α_{**}=0.01, α_{*}=0.05) using the bootsrapping test (10,000 resamples). mRNA from single cells was probed for expression of markers of pluripotency, as well as for other signalling pathways and cell fate markers, as indicated.

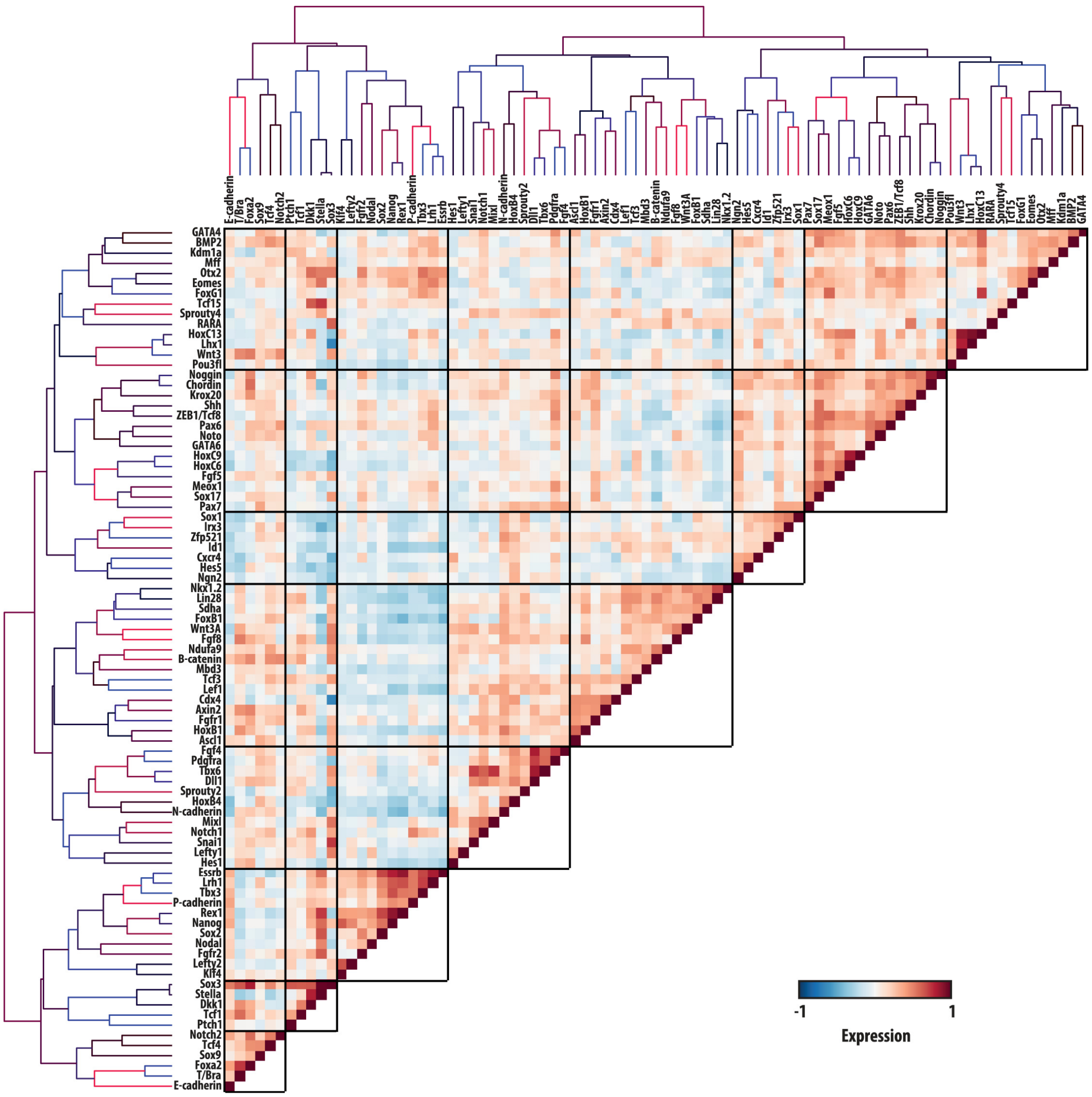

S5. Single cell analysis of the NMp population derived from mESCs. We computed all pairwise correlations between the 85 genes using the single-cell expression data from the 89 cells that passed the quality control. Hierarchical clustering of the genes according to correlations exposes the structure of concomitant genes and unveils clusters of functionally related genes. Namely, the group of genes involved in pluripotency which includes Nanog, Sox2 and Rex1; a cluster including Nkx1.2 as well as Wnt and Fgf signalling markers (Wnt3A, Lef1, Axin2, Fgff1, Fgf8); a neural cluster with Sox1, Ngn2 and Hes5, etc.

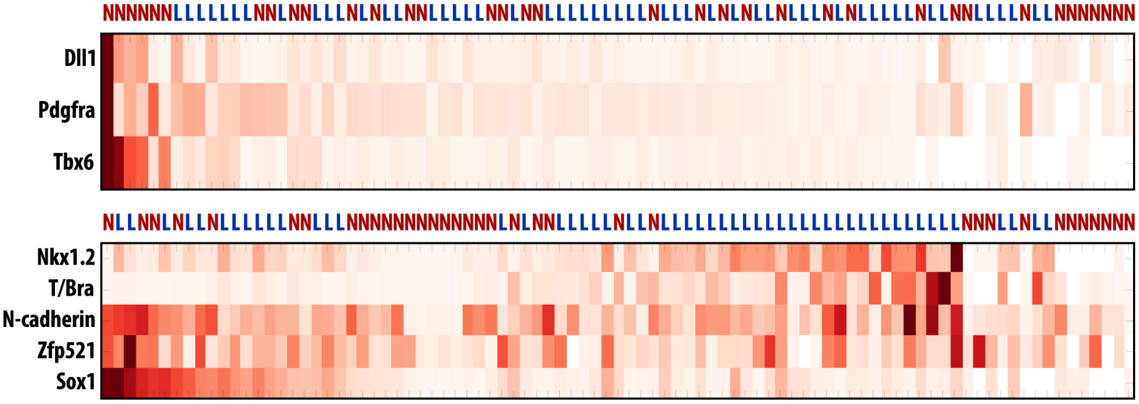

S6. Identification of cells expressing Neural or Mesendodermal markers. mRNA was extracted from Sox1::GFP single cells expressing either low (L) or negative (N) levels of the reporter and analyzed for the indicated genes. Individual cells are ordered according to the levels of Mesendodermal marker expression (top) or Neural marker expression (bottom).

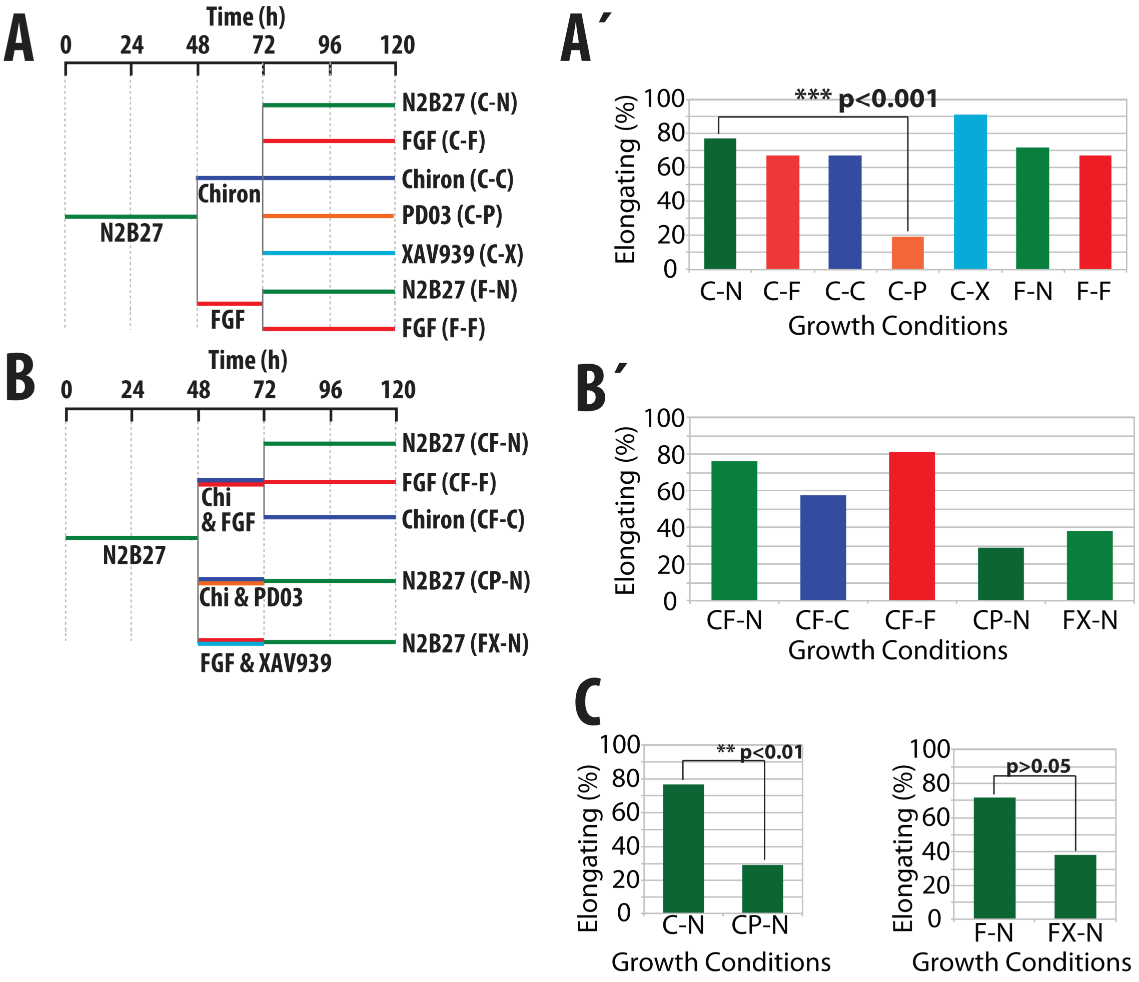

S7. Statistical analysis of morphologically scored aggregates. A test of Binomial Proportions was applied to specific pairs of conditions from the data set in Figure 9. Images of 21 aggregates were scored qualitatively for each condition and the number of elongating aggregates compared. *P* values are presented for each comparison and were assigned stars representing the degree of statistical significance (α_{***}=0.001, α_{**}=0.01, α_{*}=0.05). FGF inhibition during elongation significantly reduces the proportion of elongating aggregates (see A'). Similarly, FGF inhibition has a significant effect on the establishment of the precursor population, as shown in (C). The effect of Wnt inhibition during this period is not statistically significant, although the statistical power of the test might be limited by the small sample sizes.

Table S1: The number of cells used for the quantification of confocal images in Fig. 2B and 3C.

	Condition	Cells Analysed
Figure 2B	0-2N, 2-3 Chi	1124
	0-2B, 2-3 Chi+SB43	931
	0-2N, 2-3 Chi+PD03	1443
Figure 3C	Sox1 Negative	886
	Sox1 Low	289
	Sox1 High	459

Supplemental Table S2: Primers used for qRTPCR.

Gene Name	Forward primer sequence	Reverse primer sequence
En1	TCTCTGGGTACCTCCTGCAC	TCCAGAAAAGGAAGGGGATT
HoxB1	ATCTGGAGTCAGCCCATCCT	TCACACTCAAACGCATCCTC
HoxB8	GCGCTGTGAGCATTGTTAAA	TAACTCTTCCGCCCTTTTCA
HoxC5	CATTTCCTCTCCAGCCTTCC	CCAGAGCCACTCTGTTTCCT
HoxC6	CCCTCTCTTCTCCCTTGCTC	CCACGTCTGACTCCCTGTTT
HoxC9	AGAACCGGAGGATGAAGATG	TTTGTGTTTCCCTTGGCTGT
Nkx1.2	ACAACCACACAAGCCACTGA	CCATCCTGGGAACCCTTATT
Otx2	CTGGGCTGAACATTCCAGTT	GTCCATTTCAGGTTGCTGGT
Six3	TCCCGCTTGCTCTCTCTAAC	CCAAGCAAGTGTTTGTTTCC
Sox1	AGACAGCGTGCCTTTGATTT	TGGGATAAGACCTGGGTGAG
Sox2	CATGAGAGCAAGTACTGGCAAG	CCAACGATATCAACCTGCATGG
T/Bra	CTGGGAGCTCAGTTCTTTCG	GTCCACGAGGCTATGAGGAG
Tbx6	CACCTTGATTTCACTCCACCC	TCCCTCCATTCGCACTAAGG

Supplemental Table S3: Primers used for Fluidigm.

Gene Name	Notes	Forward primer sequence	Reverse primer sequence
Ascl1	Neural	TGCATTCCAACATCATTTCC	GTGTGTGTGACGCTCTTGCT
Axin2	Wnt	CTAGACTACGGCCATCAGGAA	GCTGGCAGACAGGACATACA
B-catenin	Wnt	GGTGGGCTGGTATCTCAGAA	CTTGTGATCCATTCGTGTGC
BMP2	Nodal BMP	TGGTTGTCCAATCCGTGAG	ACTGGTCCTGCTTTGTTCTTG
Cdx4	Primitive streak	AGACACACGATGAGTTGGAGA	CAAGAGCATATGCACAAACAAG
Chordin	Nodal BMP	GAGAAGGAGTGGTGCTGAGG	AGGAGTTCGCATGGATATGG
Cxcr4	Primitive streak	GGGTCATCAAGCAAGGATGT	GAGCCTCTGCTCATGGAGTT
Dll1	Notch	GACCATGGAGCCGAGAAGA	CCTGACCGTGGCTTCATCT
E-cadherin	Pluripotency	CAATGCCTGCTCTTGATGGT	GGGAGATCTGACTGCCTCTG
Emx1	Head	ACTTCTACCCCTGGGTGCTT	GCTCCCACCACGTAGTGATT
En1	Head	TCTCTGGGTACCTCCTGCAC	TCCAGAAAAGGAAGGGGATT
Eomes	Primitive streak	GGCTGAGCCTGAGAGTCAAG	CCAGCCCTACAACAAATGGT
Essrb	Pluripotency	GGGATCTCTTTCTCTCGTTCC	TCGTATTTCTCAAGCTCTGTCC
Fgf4		GGCCACTCCACAGAGATAGG	ACTTGGGCTCAAGCAGTAGG
Fgf5	Epi	TGGCATTATGTGGAATCTGG	CTGTGGACGCTGCACACTT
Fgf8	Primitive streak	AGGACTGCGTATTCACAGAGAT	CATGTACCAGCCCTCGTACT
Fgfr1	FGF	AGCTGTGGTTTGGGTCATTG	CCTAGCCCTCTTTGCCAAATG
Fgfr2	FGF	GCCCGGGTTACACATTCATC	CCCAGACTCTCAACCGAATG
Foxa2	Primitive streak	CATTACGCCTTCAACCACCC	GGTAGTGCATGACCTGTTCG
FoxB1	Neural	AGAAATCCTAGGGCGAGTGG	ACTCTTTGCAGCCCTTTAGC
FoxG1	Head	AACAGGCTGCCAGATTTCC	AATTCCTTGGGCACCTTTAC
GATA4	Mes/End	GCCCAGTTGTGCAGCTAATG	GGTGCAGATGAGCCATAGTC
GATA6	Mes/End	GTTGCAGCAATCAGTGTTAAATC	GAAGTGGGCTGTGAGTGTAAG
Hes1	Notch	GCCAATTTGCCTTTCTCATC	AGCCACTGGAAGGTGACACT
Hes5	Notch	CTTCTGCGAAGTTCCTGGTC	GAGGAAACACCTGCAGTTCC
HoxB1	Spinal cord/AP	ATCTGGAGTCAGCCCATCCT	TCACACTCAAACGCATCCTC
HoxB4	Spinal cord/AP	TGTCCCCTACTCCTGACACC	GTGGGTACAGACAGGGAGGA
HoxB8	Spinal cord/AP	GCGCTGTGAGCATTGTTAAA	TAACTCTTCCGCCCTTTTCA

HoxC13	Spinal cord/AP	CCCTAGCCACACCCTAACC	TTGTCCTCCTTGGATTTCCTT
HoxC6	Spinal cord/AP	CCCTCTCTTCTCCCTTGCTC	CCACGTCTGACTCCCTGTTT
HoxC9	Spinal cord/AP	AGAACCGGAGGATGAAGATG	TTTGTGTTTCCCTTGGCTGT
Id1	Primitive streak	TCCAGTGGGTAGAGGGTTTG	AATCCGAGAAGCACGAAATG
Irx3	Head	ATCTGGACGCTGCTCTGG	CAGACATGCTTGCAACTCG
Kdm1a	Housekeeping	CATGGTGCTCTGTTGAGTGG	ATGCCGTTGGATCTCTCTGT
Klf4	Pluripotency	CCAGCAAGTCAGCTTGTGAA	TTTGTAAGTCCGGGCATGTT
Krox20(Egr2)	Head	AATGGCTTGGGACTGACTTG	TGAGATGGCCAGAGAAACCT
Lef1	Wnt	GCGAATGTCGTAGCTGAGTG	GCTGTCTCTCTTTCCGTGCT
Lefty1	FGF	AGGGTGCAGACCTGTAGCTG	GGAAGCAAAGAGCACACACA
Lefty2	FGF	ACCTTAGCCCTAGACAAGAGC	AGTTTCATAGAATTCACCAGGGC
Lhx1	Primitive streak	CCAGTGGACCTACCCTTTGT	CGCTGACATGGAGTGGAGAG
Lin28	Pluripotency	CCAGGAACCTCATTCTTTCC	ACCCGAGGCTCTCTATCTCC
Lrh1	Pluripotency	GAGGGCAGAGATAGCAAACC	TTGATCCACATCTGCACAGC
Mbd3	Pluripotency	GGAAACACACCTGGCTATGC	GAAAGTGACTTCCTGGTGGG
Meox1	Primitive streak	GCACAAGAGCTGATGGATGA	ACGCAGGATAGGTCCAAATG
Mesp1	Primitive streak	CGCCTGCCTACCCTAGACC	CTGCTGAAGAGCGGAGATG
Mff	Housekeeping	TAACAGCTGGCTCTGGTTTC	CACCACAGGCCTTCACTCTAC
Mixl1	Primitive streak	GGCAGCTTCCAGTTAACCAA	CTGAGTCCCAACCAGAAAGG
Msgn	Primitive streak	GGCCTCAAAGGCCAGAAAG	GAGGGCATCGGCTAAGGT
Nanog	Pluripotency	CTTTCACCTATTAAGGTGCTTGC	TGGCATCGGTTCATCATGGTAC
N-cadherin	Neural	GGGATGAGACCACAAGATAGGA	AAACTCCCTTTATCTGCAACCA
Ndufa9	Housekeeping	GATCCAGATGCCGTAGGAAA	AAATGGACTCAGCCCAAAGA
Ngn2	Neural	GCAACTGGTCCCTGTGATCT	ACAGGTGAAATTCCCACAGC
Nkx1.2	Neuromuscular progenitor	ACAACCACACAAGCCACTGA	CCATCCTGGGAACCCTTATT
Nodal	Nodal BMP	AGCCACTGTCCAGTTCTCCAG	GTGTCTGCCAAGCATACATCTC
Noggin	Neural	CCCATCATTTCCGAGTGTAAG	CTCGCTAGAGGGTGGTGAAA
Notch1	Notch	TCCAATGTGCATTGTGGACT	TGCAAGAATCTGCTGTGAGC
Notch2	Notch	CTGACTTATGCGATGGTGGG	ATGCAAGACTTCAAGTGGCC
Noto	Spinal cord/AP	CAGCTGGTGAGGTTTATGGG	TGATGCTGATTCAAGGCTGC
Oct4	Pluripotency	GATGCTGTGAGCCAAGGCAAG	GGCTCCTGATCAACAGCATCAC

Otx2	Head	CTGGGCTGAACATTCCAGTT	GTCCATTTCAGGTTGCTGGT
Pax6	Neural	AAGCACTTCACTTTGTAACTGTCC	CCAACTGATACCGTGCCTTC
Pax7	Neural	CAAGGTCTGGACAAGAGGAAAG	GAGCAAGGAATGTGGAGGAG
P-cadherin	Pluripotency	TCGATTCAAGAAACTGGCGG	CTTGAGATGCTGCTGTGACC
Pgdfra	Mes/End	CCACACTCAGCAGGGATACA	GGGCAAGCTTTGATTGTCAT
Pou3fl	Neural	CTCTCCTCCTTCAAGAACGC	CTCTGCTAGGCCAGGAACC
Ppia	Housekeeping	TTACCCATCAAACCATTCCTTCTG	AACCCAAAGAACTTCAGTGAGAGC
Ptch1	Hedgehog	GGAGCTCAGGCAATACGAAG	GGTCAAGGGAGGCTGATGT
RARA	FGF	CTGGATCTCGAGCTGAAGGG	ACTGAGGAGCAAAGACTGGG
Rex1	Pluripotency	ACTGTGGTGTCTTATCGATGCT	TGCCACACTCTGCACACAC
Sdha	Housekeeping	CCGCTCCTACTGATGAAACC	AAGTCTGGCGCAACTCAATC
Shh	Hedgehog	CGAGGATGGAGCCTGTAGTT	TGTGTGGCACGCTTTATTTC
Six3	Head	TCCCGCTTGCTCTCTCTAAC	CCAAGCAAGTGTTTGTTTCC
Snai1	Primitive streak	AGCCAGACTCTTGGTGCTTG	ACCCACTCGGATGTGAAGAG
Sox1	Neural	AGACAGCGTGCCTTTGATTT	TGGGATAAGACCTGGGTGAG
Sox17	Mes/End	TTCTGTACACTTTAATGAGGCTGTTC	TTGTGGGAAGTGGGATCAAG
Sox2	Neural	CATGAGAGCAAGTACTGGCAAG	CCAACGATATCAACCTGCATGG
Sox3	Neural	CTGACCCACATCTGAGCTCC	TTCGGTCCTCCTCATCTTCG
Sox9	Neural	TCTGCCTGGACTGTATGTGG	TCTGTCCGATGTCTCTCTGC
Sprouty2	FGF	ACAATTCAGCTAATGGAACCCG	TCTTCGCCTAGGAGTGTTGG
Sprouty4	FGF	ATGGTGGATGTCGATCCTGT	GGAGGGGGAGCTACAGAGAC
Stella	Pluripotency	ATCCGGAGGGAAGTTCAAAG	TCCCGTTCAAACTCATTTCC
T/Bra	Primitive streak	CTGGGAGCTCAGTTCTTTCG	GTCCACGAGGCTATGAGGAG
Tbx3	Pluripotency	GCAGCTATGGGCATGAAAC	TAATGGTCCAACAGGCACAC
Tbx6	Mes/End	CACCTTGATTTCACTCCACCC	TCCCTCCATTCGCACTAAGG
Tcf1	Wnt	TGCAAGAGGAGGGACTAGGA	TGGGAAGAGCCTGATAGGAA
Tcf15	Epi	CACTCCTGCGTTGTGTAAGG	CTGGATGGCTAGATGGGTCC
Tcf3	Wnt	ACGGTTCTGGATGAGACAGG	AAGCAGGGAGCTGTTCAGTG
Tcf4	Wnt	TTTCAATGTGTGGCTGAGTG	GTGGGCCTCTTGAAGGAAGT
Wnt3	Wnt	CTAATGCTGGCTTGACGAGG	ACATGGTAGAGAGTGCAGGC
Wnt3A	Wnt	CATACAGGAGTGTGCCTGGA	AATCCAGTGGTGGGTGGATA

ZEB1/TCF8	Neuromuscular progenitor	ACCTGACCTGCTGTCGTTCT	AAAGGCATTCTTTGTGCTAAGTG
ZEB2/Sip1	Neuromuscular progenitor	CCATCTTGATGGCGCTTAC	TCAGGTTGGAACTGTGTTTCTC
Zpf521	Neural	GCGTCGTTCCAAAGACAAAG	GGAGATGAAGCCATTTGGAG
Dkk1	Wnt	CCATTCTGGCCAACTCTTTC	CATTCCCTCCCTTCCAATAAC
